# Shannon entropy of brain functional complex networks under the influence of the psychedelic Ayahuasca

**DOI:** 10.1038/s41598-017-06854-0

**Published:** 2017-08-07

**Authors:** A. Viol, Fernanda Palhano-Fontes, Heloisa Onias, Draulio B. de Araujo, G. M. Viswanathan

**Affiliations:** 1 Department of Physics, Universidade Federal do Rio Grande do Norte, 59078-970 Natal, RN Brazil; 2 Computational Biology Center, T. J. Watson Research Center, IBM, 10598 Yorktown Heights, NY USA; 3 Department of Physics, Universidade Federal de Viçosa, 36570-000 Viçosa, MG Brazil; 4 Brain Institute, Universidade Federal do Rio Grande do Norte, 59078-970 Natal, RN Brazil; 5 National Institute of Science and Technology of Complex Systems Universidade Federal do Rio Grande do Norte, 59078-970 Natal, RN Brazil

## Abstract

The entropic brain hypothesis holds that the key facts concerning psychedelics are partially explained in terms of increased entropy of the brain’s functional connectivity. Ayahuasca is a psychedelic beverage of Amazonian indigenous origin with legal status in Brazil in religious and scientific settings. In this context, we use tools and concepts from the theory of complex networks to analyze resting state fMRI data of the brains of human subjects under two distinct conditions: (i) under ordinary waking state and (ii) in an altered state of consciousness induced by ingestion of Ayahuasca. We report an increase in the Shannon entropy of the degree distribution of the networks subsequent to Ayahuasca ingestion. We also find increased local and decreased global network integration. Our results are broadly consistent with the entropic brain hypothesis. Finally, we discuss our findings in the context of descriptions of “mind-expansion” frequently seen in self-reports of users of psychedelic drugs.

## Introduction

Relatively little is known about how exactly psychedelics act on human functional brain networks. During the last few years, new neuroimaging techniques, such as functional magnetic resonance imaging (fMRI)^1, 2^, have allowed noninvasive investigation of global brain activity in a variety of conditions, e.g., under anaesthesia, sleep, coma, and in altered states of consciousness induced by psychedelic drugs^3–10^. Recently, Carhart-Harris *et al*. proposed a hypothesis known as the *entropic brain*, which holds that the stylized facts concerning altered states of consciousness induced by psychedelics can be partially explained in terms of higher entropy of the brain’s functional connectivity^11^. Although until recently the entropy of the brain had never been directly measured, the entropic brain hypothesis is empirically supported by several recent studies. For example, Sarasso *et al*. have reported complex spatiotemporal cortical activation pattern during anesthesia with ketamine, which can induce vivid experiences (“ketamine dreams”)^12^. Similarly, Petri *et al*. found that after administration of the psychedelic psilocybin, the brain’s functional patterns undergo a dramatic change characterized by the appearance of many transient low-stability structures^13^. Perhaps the most convincing evidence supporting the hypothesis thus far has come from the study undertaken by Tagliazucchi *et al*.^14^, who reported a larger repertoire of brain dynamical states during the psychedelic experience with psilocybin. They inferred an increase in the entropy of the functional connectivity in some regions of the brain, by studying the temporal evolution (i.e., dynamics) of the connectivity graphs. Similarly, Lebedev *et al*.^15^ and separately Schartner *et al*.^16^ have very recently reported evidence of increases in specific measures of entropy, the former with LSD and the latter with LSD, psilocybin and ketamine. Here we directly measure increases in entropy associated with the functional connectivity of the whole brain under the influence of a psychedelic. Specifically, we analyze fMRI functional connectivity of human subjects before and after they ingest the psychoactive brew Ayahuasca and report an increase in the Shannon entropy. This is the first time that the entropy of the functional networks of the human brain has been directly measured in altered states of consciousness on a global scale, i.e. considering the entire brain.

Ayahuasca is a beverage of Amazonian indigenous origin and has legal status in Brazil in religious and scientific settings^17^. It contains the powerful psychedelic *N*,*N*- dimethyltryptamine (DMT), together with harmala alkaloids that are known to be monoamine oxidase inhibitors (MAOIs). The beverage is typically obtained by decoction of two plants from the Amazonian flora: the bush *Psychotria viridis*, that contains DMT, and the liana *Banisteriopsis caapi*, that contains MAOIs^18^. DMT is usually rapidly metabolized by monoamine oxidase (MAO), but the presence of MAOI allows DMT to cross the blood-brain barrier and to exert its effects^19–24^. Similar to LSD, mescaline and psilocybin^19–24^, Ayahuasca can cause profound changes of perception and cognition, with users reporting increase of awareness, flexible thoughts, insights, disintegration of the self, and attentiveness^19, 25^. There is growing interest in Ayahuasca, partially due to recent findings showing that it may be effective in treating mental disorders, such as depression^26, 27^ and behavioral addiction^28, 29^. Similar therapeutic potential has also been pointed out for other psychedelics^9, 23, 30–33^.

For analysis, we use tools and concepts from the field of complex networks, a brief history of which follows. The application of graph theory to phase transitions and complex systems led to significant progress in understanding a variety of cooperative phenomena over a period of several decades. In the 1960s, the books by Harary, especially *Graph Theory and Theoretical Physics* ^34^, introduced readers to powerful mathematical techniques. The chapter by Kastelyn, still considered to be a classic, showed that difficult combinatorial problems of exact enumeration could be attacked via graph theory, including the exact solution of the two-dimensional Ising model (e.g., see Feynman^35^). In the 1980s, certain families of neural network models were shown to be equivalent to Ising systems, e.g., the Hopfield network^36^ is a content-addressable memory which is isomorphic to a generalized Ising model^37^. Beginning in the 1990s, new approaches to networks, giving emphasis to concepts such as the node degree distribution, clustering, assortativity, small-worldliness; and network efficiencies, led eventually to what has become the new field of complex networks^38, 39^. These new tools and concepts^40–42^ have found successful application in the study of diverse phenomena, such as air transportation networks^43^, terrorist networks^44^, gene regulatory networks^45^, and functional brain networks^46–50^. We approach the human brain from this perspective of complex networks^51, 52^.

Ten healthy volunteers were submitted to two distinct fMRI scanning sessions: (i) before and (ii) 40 minutes after Ayahuasca intake, when the subjective effects become noticeable (the volunteers drank 2.2 mL/kg of body weight and the Ayahuasca contained 0.8 mg/mL of DMT and 0.21 mg/mL of harmine, see Methods section). In both cases, participants were instructed to close their eyes and remain awake and at rest, without performing any task. See Methods for details concerning data acquisition and preprocessing.

Data analysis consists of two main steps. In the first step, we use fMRI data to generate complex networks to represent the actual functional brain connectivity patterns. In the second step, we use the networks generated in step 1 as inputs and calculate network characteristics as output, using techniques from the theory of complex networks. The Methods section describes both steps in detail. More information about most of the methods used here can be found in refs 4, 53, 54.

**Figure 1:**
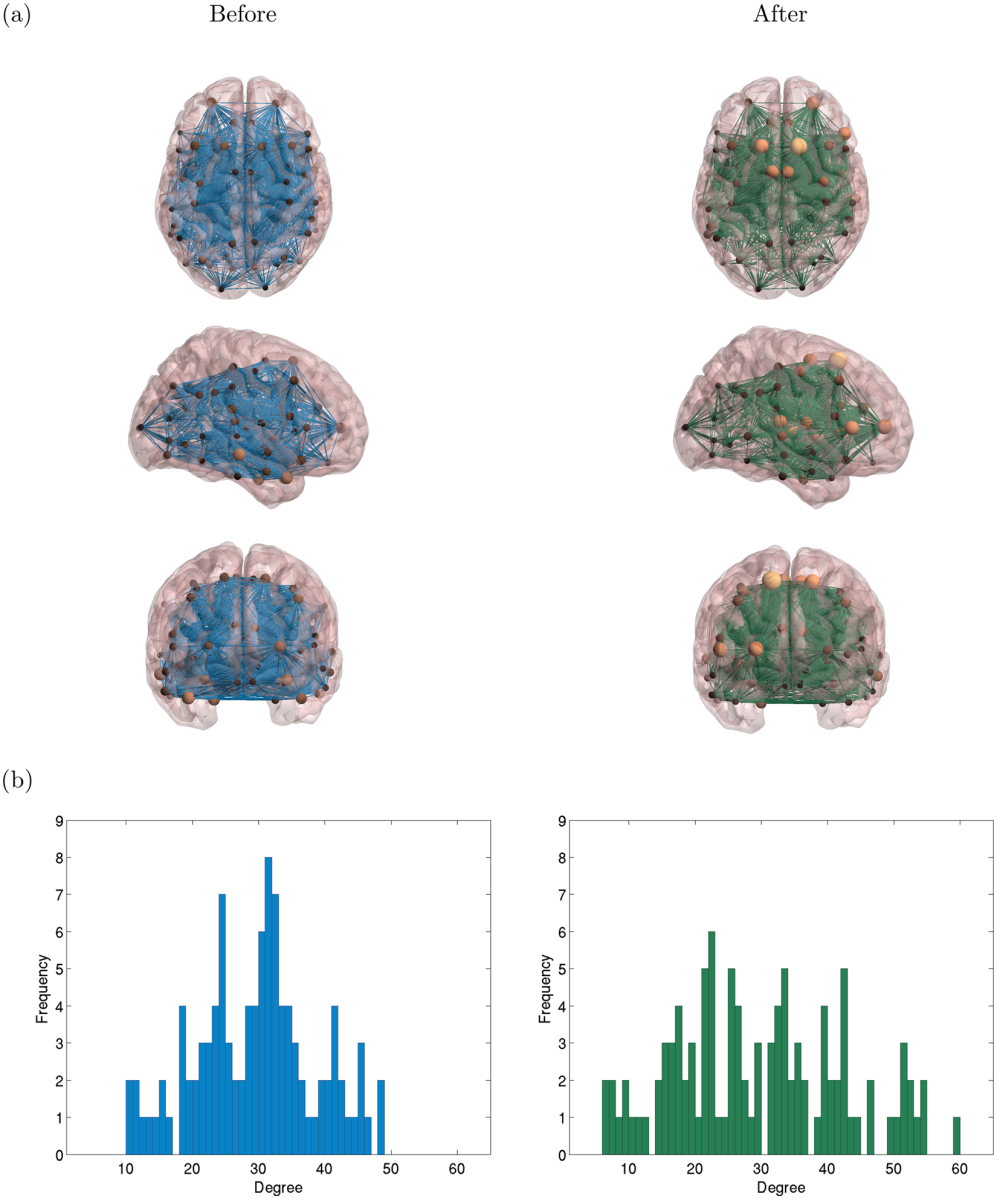
Illustrative example of functional brain networks. (**a**) 3 views of a complex network generated from fMRI data from one subject, before (left) and after (right) Ayahuasca ingestion (mean node degree 〈*k*〉 = 30). The spheres represent nodes and sphere size is proportional to the node degree. (**b**) histograms of node degrees, corresponding to the networks shown in (**a**). After Ayahuasca intake, the distribution is wider, indicating a higher entropy. In (**a**) we have used the BrainNet Viewer (http://www.nitrc.org/projects/bnv) for visualization.

Figure 1 shows the networks generated from one subject before and after Ayahuasca intake, for one specific choice of mean node degree. The spheres represent nodes, with sphere size proportional to the degree of the node. The lower plots show histograms of node degrees.

The main result that we report here is an increase in the Shannon entropy of the degree distribution for the functional brain networks subsequent to Ayahuasca ingestion. We also find that the geodesic distance increases during the effects of Ayahuasca, i.e. qualitatively the network becomes “larger”. More generally, we also find that these functional brain networks become less connected globally but more connected locally.

The key technical innovation is the measurement of the Shannon entropy of the degree distribution of the complex networks that represent the functional connectivity of the human brain. Moreover, the Shannon entropy is also very closely related to the Boltzmann-Gibbs entropy used in statistical mechanics. Hence, our approach to studying the brain experimentally is grounded in two strong theoretical traditions: graph theory and complex networks on the one hand, and information theory and statistical physics on the other. Our study also represents a significant advance for the following additional reasons: (i) our results unveil how Ayahuasca (and likely most other tryptamine psychedelics) alter brain function, both locally and globally; (ii) it is the first time this specific approach has been applied to characterize functional brain networks in altered states of consciousness; (iii) our study of Ayahuasca covers all brain regions; and (vi) the method we have developed can be immediately applied to study a variety of other phenomena (e.g., the effects of medication for mental health disorders).

## Results

### Increase of the Shannon entropy of the degree distributions

**Figure 2:**
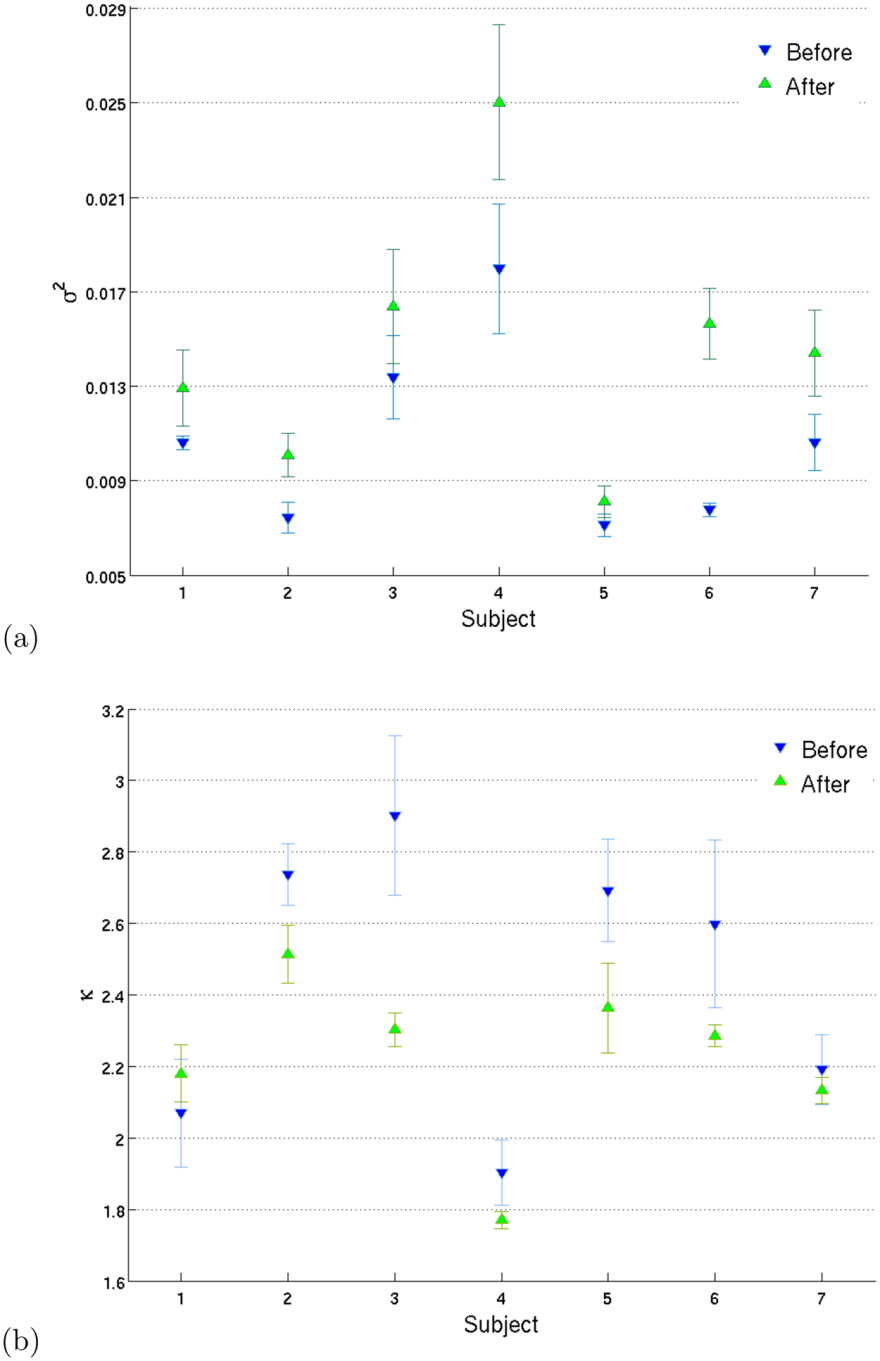
Variance and kurtosis of the degree distribution. Mean ± 1 standard deviation calculated over all 16 networks of the degree variance (**a**) and kurtosis (**b**), shown for each subject (blue ∇) and after (green Δ) Ayahuasca ingestion. The individual values for the degree variance and kurtosis are calculated separately for each network. We find higher variance and (mostly) lower kurtosis after Ayahuasca, hence the node distributions change shape and become less “peaked”. Such behavior is again consistent with (if not suggestive of) a higher Shannon entropy after Ayahuasca.

We find evidence of significant changes in the functional brain networks of subjects before and after ingestion of Ayahuasca. Figure 2 shows 2nd as well as 4th central moments of the degree distributions for each subject. The individual values are calculated separately for each network. We find an increase of variance for all subjects after Ayahuasca intake and a decrease of kurtosis for almost all of them (6 subjects). These findings indicate that the degree distributions become less peaked and wider. This behavior is suggestive of an increase of the Shannon entropy for the degree distributions after Ayahuasca ingestion.

**Figure 3:**
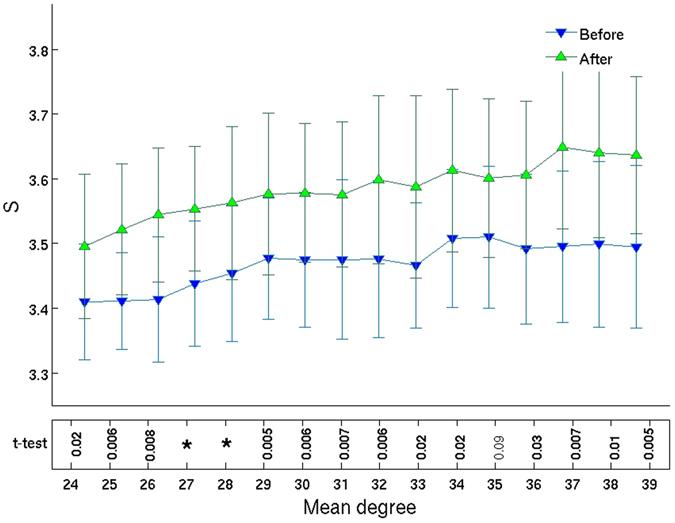
Entropy grows after Ayahuasca ingestion. Mean ± 1 standard deviation of the Shannon entropy of the distribution of node degrees, calculated over all 7 subjects, as a function of mean degree *k*, before (blue ∇) and after (green Δ) Ayahuasca intake. The bottom row lists *p*-values for Student’s paired *t*-test, with values *p* < 0.005 indicated by asterisks (*). (Confidence intervals are shown in Supplementary Figure S5). We thus see evidence against the null hypothesis of no change in entropy. Indeed, we find a significant increase in the entropy of the degree distributions after Ayahuasca ingestion. This entropy increase is the main result that we report.

**Figure 4:**
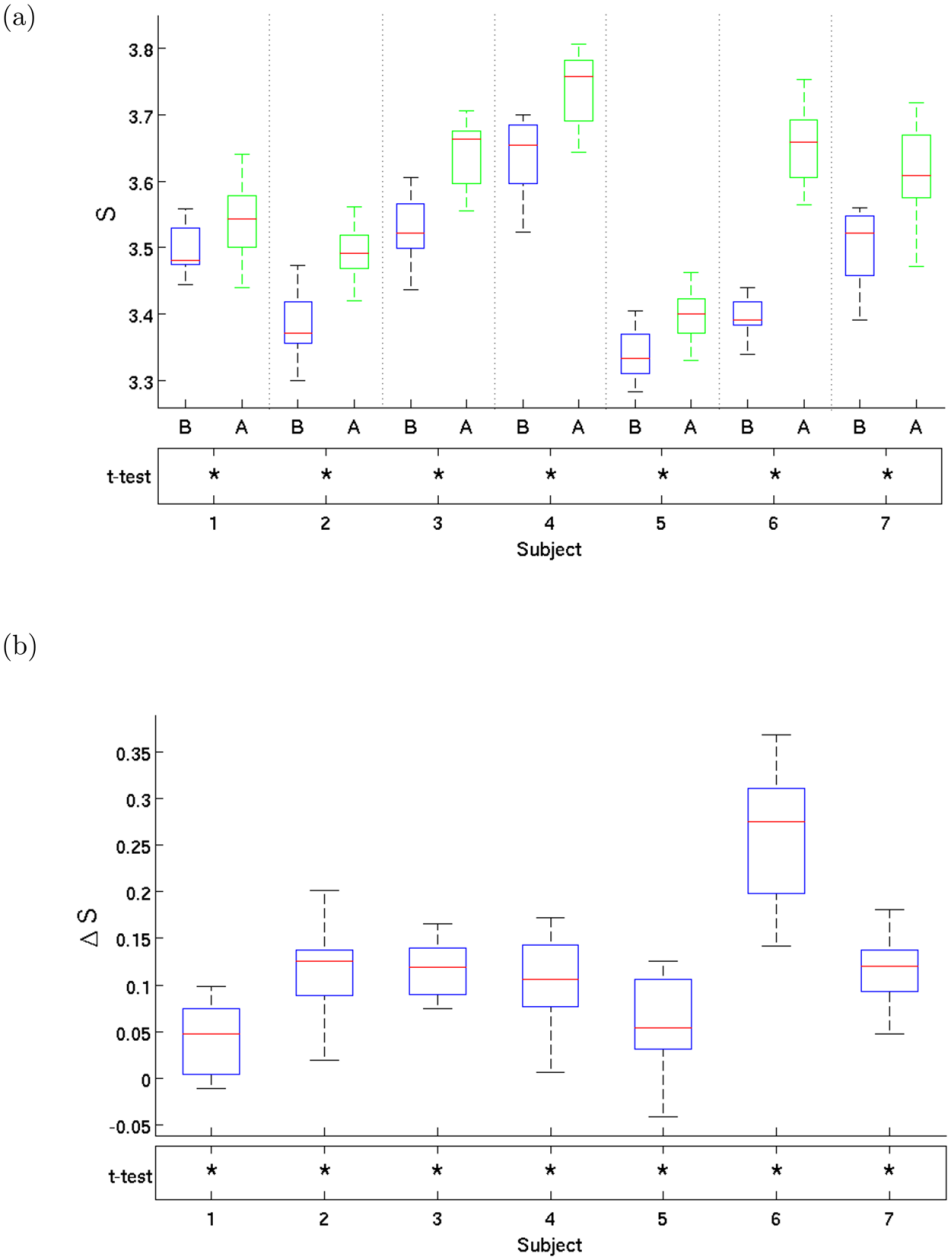
Entropy growth per subject. (**a**) Boxplot of the entropy distribution and before (**B**) and after (**A**) Ayahuasca ingestion and (**b**) boxplot of entropy increase, for all 7 subjects. Note the significant increase in entropy after Ayahuasca ingestion. There are 16 values of entropy per subject, as discussed in the text. The bars show minimum and maximum values and the box shows the 2nd and 3th quartiles, with the median shown dividing the box (in red). The asterisks (*) in the bottom rows in both plots indicate *p*-values *p* < 0.005 for Student’s paired *t*-test in (**a**) and *t*-test for zero mean in (**b**). Subject-by-subject, we thus find strong evidence against the null hypothesis of no entropy change.

Figure 3 shows the average Shannon entropy of the degree distributions as a function of mean degree, considering networks from all subjects, before and after Ayahuasca intake. A fair comparison of the “before” and “after” networks is possible by considering the entropy of networks of identical mean degree. We find an increase in the entropy of the degree distributions after Ayahuasca ingestion. In order to better evaluate the consistency of this result, we also calculate the average Shannon entropy subject-by-subject, before and after Ayahuasca (Fig. 4). We find significant increased entropy for all individual subjects.

To ensure that the observed changes in entropy are not due to augmented motion of subjects’ heads during the effects of Ayahuasca, we also study the correlation between changes in individual Frame Displacement (FD)^55^ against individual changes in entropy. We find a Pearson correlation coefficient of 0.005 (hence, *p*-value ≈ 1), between entropy increases and motion (see Supplementary Figure S1). Hence, we discard the possibility that the observed entropy changes are due to augmented motion.

### Iso-entropic randomized networks

The degree distribution does not completely define a network, however it can have great influence over other network properties. One can quantify this influence by comparing any given network *G* to other networks chosen randomly from the ensemble of networks that have exactly the same degree distribution. We refer to such networks as “randomized networks”. By definition, all such randomized networks have the same entropy as the original network *G*, i.e. they are iso-entropic to *G*.

An efficient way of generating such randomized networks is the Maslov algorithm^56^ (see Methods). Whereas entropy is conserved by the Maslov algorithm, the clustering coefficient, geodesic distances and efficiencies are not. By comparing these non-conserved quantities before and after randomization, we can distinguish effects that are due solely to changes in the degree distribution from those that are sensitive to how links are more specifically arranged.

We generate a set of 30 iso-entropic randomized networks for each original network, for all subjects both before and after Ayahuasca ingestion. Comparison of the original networks with the randomized networks yields important information concerning to what degree the changes in quantities such as geodesic distance, clustering coefficients, and global and local efficiencies can be accounted for by the changes in the degree distributions (see results described below).

### Decrease of global integration

**Figure 5:**
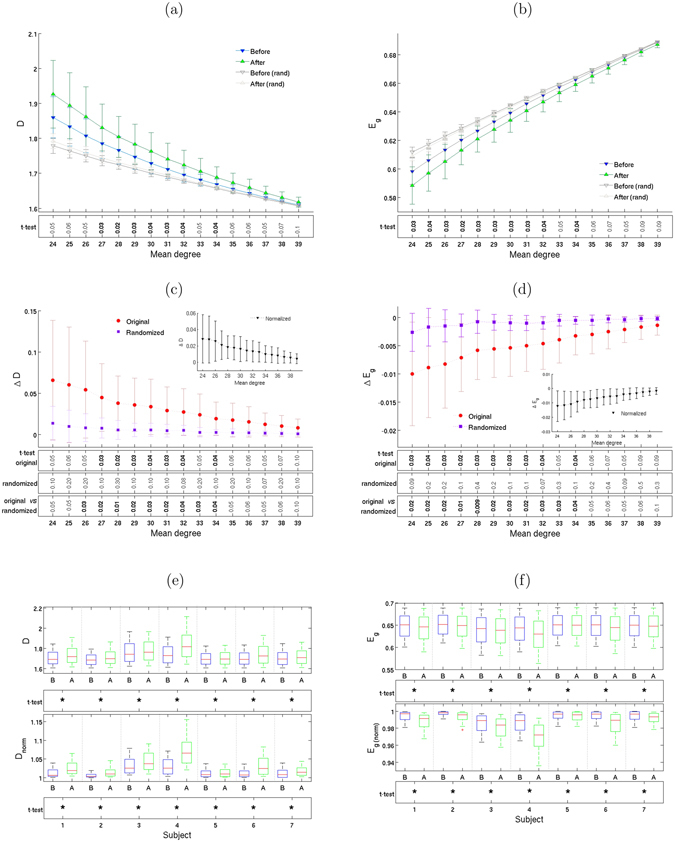
Global efficiency and integration decrease. Geodesic distance *D* (left column) and global efficiency *E*_*g*_ (right column). Plots (**a**) and (**b**) show means ± 1 standard deviations, calculated from the complex networks of all 7 subjects, as well as from their corresponding iso-entropic randomized networks, for 16 different mean degrees. Plots (**c**) and (**d**) show the change in *D* and *E*_*g*_ after Ayahuasca ingestion. The inset shows normalized values (see text). Boxplots (**e**) and (**f**) show the same information, subject-by-subject. As in previous figures, the rows below the plots show *p*-values for the *t*-test, with asterisks (*) indicating *p* < 0.005.

Figure 5 shows an increase of mean geodesic distance and a decrease of global efficiency after Ayahuasca ingestion. To determine how much of the change in geodesic distance is due to the change in the degree distribution, we also calculated the geodesic distance and global efficiency for the iso-entropic randomized networks. Note how the values for those networks are quite different compared to the non-randomized networks. We conclude that the change in degree distribution cannot explain the entire change in geodesic distance. The inset in the middle panels shows the change in the normalized mean geodesic distance and global efficiency, which we define as the ratio *D*/*D*_rand_ and similarly for the global efficiency (see refs 4, 53). We see, indeed, that these ratios are not close to zero. If the change in degree distribution could account for all the change in geodesic distance and efficiency, then the change in these ratios would be close to zero. Significant changes are also observed at the individual level and are again consistent for all subjects (Fig. 5(e) and (f)).

### Increase of local integration

**Figure 6:**
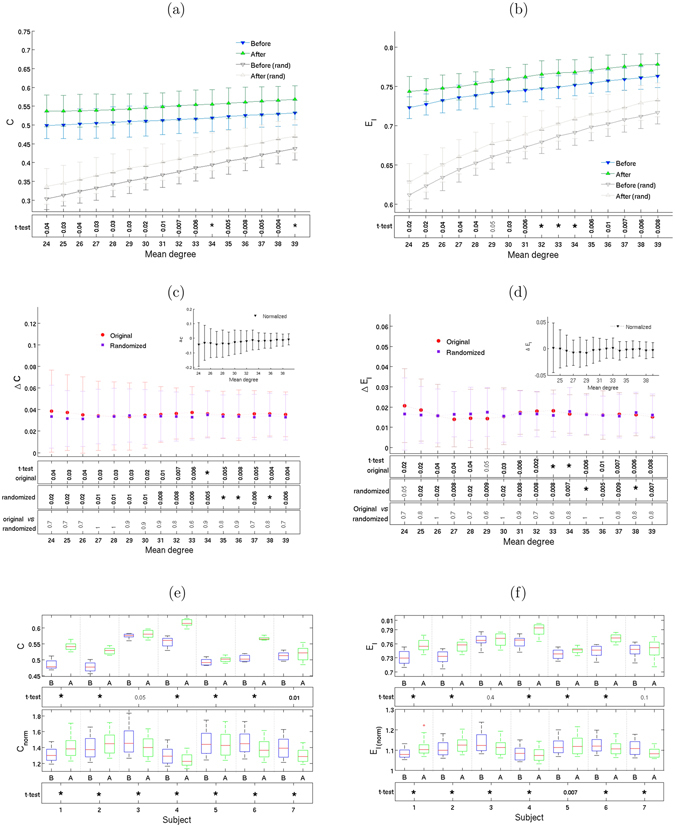
Local efficiency and integration increase. Clustering coefficient *C* (right column) and local efficiency *E*_*l*_ (left column). Plots (**a**) and (**b**) show means ± 1 standard deviations, calculated from the complex networks of all 7 subjects, as well as from their corresponding iso-entropic randomized networks, for 16 different mean degrees. Plots (**c**) and (**d**) show the change in *C* and *E*_*l*_ after Ayahuasca ingestion. The inset shows normalized values (see text). Boxplots (**e**) and (**f**) show the same information, subject-by-subject. As before, the rows below the plots show *p*-values for the *t*-test, with asterisks (*) indicating *p* < 0.005.

Figure 6 shows an increase of clustering coefficients and local efficiency after Ayahuasca ingestion. In contrast to the behavior of the metrics discussed above, almost identical changes are seen for iso-entropic networks. This result indicates that the variation in degree distribution can account for most of the change in clustering and local efficiency. The insets in the middle panel show the change in the normalized clustering and local efficiency, which we define as the ratio *C*/*C*_rand_ and similarly for the local efficiency. We see, indeed, that these ratios are close to zero.

### Subjective evaluation

The subjective states of the subjects were evaluated using two psychometric scales: the Clinician Administered Dissociative States Scale (CADSS)^57^ and the Brief Psychiatric Rating Scale (BPRS)^58^. We report a significant correlation (*r* = 0.91; *p* = 0.004) between individual CADSS scores variation and the entropy before Ayahuasca intake (see Supplementary Fig. S2), however one must proceed with caution when interpreting these findigns because of the small number of subjects. The correlations between CADSS score variation and the variation in geodesic distance and global efficiency reach *r* = 0.71 and *r* = 0.67, respectively. Although these correlations fall short of our significance criterion (*r* > 0.76, *p* < 0.05), yet these *r* values are relatively large when compared with those for the clustering coeficient (*r* = 0.15) and local efficiency (*r* = 0.09).

## Discussion

Our results reveal that the entropy increases after Ayahuasca ingestion. The following also increase: geodesic distance, clustering coefficient and local efficiency. However, the global efficiency decreases. Overall, we find an increase of local integration and a decrease of global integration in the functional brain networks.

We interpret these findings in the context of some well understood prototypical classes of networks. Regular lattices have fixed coordination number, hence all nodes have the same degree and the Shannon entropy of the degree distribution is thus zero. In contrast, the entropy is high in networks with broad distributions of degree. The observed increase in entropy after Ayahuasca ingestion indicates that the degree distribution becomes broader. In the context of the Watts-Strogatz model^59^, clustering and geodesic distance both decrease when highly regular networks are transformed into small-world networks by randomly re-assigning the links. Whereas clustering and geodesic distances decrease with increasing randomness in such models, we find the opposite behavior for Ayahuasca, i.e., randomness as measured by the Shannon entropy of the node degree distribution increases in parallel with clustering and geodesic distances. Hence, our findings cannot be reduced to simple explanations of greater or lesser randomness. Locally, there is an increase in integration (as measured by network efficiency), but globally there is a decrease in integration. Indeed the increase of geodesic distance and decrease of global efficiency after Ayahuasca intake signify that the functional brain networks are less globally integrated. One possible interpretation of these findings is that the increase of local robustness and the decrease of global integration reflect a variation in modular structure of the network. Recent studies have reported the presence of modularity in functional brain networks on several scales^50, 60, 61^. Modular networks are characterized by the existence of reasonably well-defined subnetworks in which internal connections are denser than connections between distinct subnetworks^50^. However, traditional algorithms^62–64^ were not able to detect variation on modular structure features between our sets of networks.

Our results are broadly consistent with the entropic brain hypothesis, hence we discuss the latter in the context of our findings. The hypothesis maintains that the mental state induced by psychedelics, which the original authors term “primary-state,” presents relatively elevated entropy in some features of brain organization, compared to the ordinary waking state (termed “secondary”)^11^. Although it may be somewhat counter-intuitive that the psychedelic state is considered primary while ordinary consciousness is secondary, their hypothesis is inherently plausible considering that a wider spectrum of experiences is possible with psychedelics than in ordinary consciousness. In this sense, ordinary consciousness can be thought of as a restriction or constrained special case of a more primary consciousness. The hypothesized lower entropy of ordinary consciousness relative to primary consciousness is attributed to this reduction of freedom. In fact, the idea that ordinary consciousness is not primary was previously put forth by Alan Watts to describe what later became widely known as *mindfulness* ^65^. (We emphasize that the terms “primary” and “secondary” are used not as value judgements, but rather to denote temporal order, i.e. the order of appearance, both evolutionarily as well as in the maturation of a healthy individual).

More specifically, we believe that the observed increase in entropy may be related to the temporary removal of the some of the restrictions that are necessary for sustaining ordinary (adult trained) consciousness. With these restrictions temporarily gone, the entropy increases and the mind may become effectively more “free”, attaining a more flexible state in which self-referential narratives and thoughts about the past or the future are no longer mentally identified with the reality that they represent. We emphasize, however, that “correlation is not causation” and that the entropy increases may occurr in parallel with the loss of constraints, rather than bear a direct causal relation to them. These ideas are further explored in the Supplementary Discussion (see also refs. [80–83]).

Relatively few studies have investigated entropy in brain functional networks, hence additional comments are in order. Tagliazucchi *et al*.^14^ showed that psilocybin (psychedelic present in some species of mushrooms) may be responsible for increases of a different entropy measure in functional connectivity of the 4 regions of Default Mode Network (DMN), a relevant functional network related to resting state. Recently, Yao *et al*.^66^ correlated entropy increases in the human brain with age. This study also supports the view that entropy is correlated to brain function (and perhaps also its development). Moreover, in agreement with our results, a study by Schröter *et al*.^4^ similarly suggests that functional network topology may have a central role in consciousness quality. They investigated the effects on the human brain of the anesthetic propofol, which can induce loss of consciousness^67^. They reported a decrease of the clustering coefficient, which is strongly influenced by degree distribution (however, geodesic distance remained unchanged). Very recently, Lebedev *et al*.^15^ and separately Schartner *et al*.^16^ have reported evidence of increases in measures of entropy, the former with LSD and the latter with LSD, psilocybin and ketamine.

We briefly comment on the limitations of our method: (i) the reduced number of subjects and the fact that all of them were experienced with Ayahuasca do not allow population inferences and do not elucidate whether the effects observed here were only due the acute administration or if previous experience also played a significant role; (ii) expectancy and suggestion were not controlled, as placebo was not used; (iii) networks were built upon a number of critical choices, such as the atlas used to partition the brain, the method used to build the correlation matrix, and the cutoff thresholds for generating the adjacency matrices from correlation matrices^68, 69^, which may affect the final results; (iv) the chosen range of correlation values automatically limits the networks’ behavior to a small-world network. Despite this limitation, it is important to highlight that several studies have consistently demonstrated that brain networks exhibit a small-world behavior^70^.

Finally, we speculate about whether or not our finding of larger mean geodesic distances may have any relation to self-reports of “mind-expansion” by users of psychedelics (see also ref. 16). Could there be a direct relation between entropy increases and the higher creativity reported by users of psychedelics? Such questions merit further investigation. In conclusion, our results are broadly consistent with the hypothesis that psychedelics increase the entropy in brain functions. By calculating the Shannon entropy of the degree distribution of complex networks generated from fMRI data, we have taken a new low-computational-cost approach to investigating brain function under the influence of psychedelics.

## Methods

### Data acquisition and preprocessing

The fMRI images were obtained in a 1.5 T scanner (Siemens, Magneton Vision), using an EPI-BOLD like sequence comprising 150 volumes, with the following parameters: TR = 1700 ms; TE = 66 ms; FOV = 220 mm; matrix 64 × 64; voxel dimensions of 1.72 mm × 1.72 mm × 1.72 mm. It also was acquired whole brain high resolution T1-weighted images (156 contiguous sagittal slices) using a multiplanar reconstructed gradient-echo sequence, with the following parameters: TR = 9.7 ms; TE = 44 ms; flip angle 12°; matrix 256 × 256; FOV = 256 mm, voxel size = 1 *mm* × 1 *mm* × 1 *mm*. The images were obtained from 10 healthy right-handed adult volunteers (mean age 31.3, from 24 to 47 years), all who were experienced users of Ayahuasca with at least 5 years use (twice a month) and at least 8 years of formal education. The experimental procedure was approved by the Ethics and Research Committee of the University of São Paulo at Ribeirão Preto (process number 14672/2006). Written informed consent was obtained from all volunteers, who belonged to the Santo Daime religious organization. All experimental procedures were performed in accordance with the relevant guidelines and regulations.

Volunteers were not under medication for at least 3 months prior to the scanning session and were abstinent from caffeine, nicotine and alcohol prior to the acquisition. They had no history of neurological or psychiatric disorders, as assessed by DSM-IV structured interview^71^. Subjects ingested 120–200 mL (2.2 mL/kg of body weight) of Ayahuasca known to contain 0.8 mg/mL of DMT and 0.21 mg/mL of harmine. Harmaline was not detected via the chromatography analysis, at the threshold of 0.02 mg/mL^7^. Preprocessing steps were conducted in FSL (http://www.ndcn.ox.ac.uk/divisions/fmrib) and include: slice-timing correction, head motion correction and spatial smoothing (Gaussian kernel, FWHM = 5 mm). One volunteer was excluded from analysis due to excessive head movement (more than 3 mm in some direction), leaving 9 participants (5 women) to our analysis. All images were spatially normalized to the Montreal Neurologic Institute (MNI152)^72^ standard space, using a linear transformation. We also evaluated 9 regressors of non-interest using a General Linear Model (GLM): 6 regressors to movement correction, 1 to white matter signal, 1 to cerebrospinal fluid and 1 to global signal. Each volunteer was submitted to fMRI scanning under two distinct conditions: (i) before and (ii) 40 minutes subsequent to Ayahuasca intake. In both cases, volunteers were in an awake resting state: they were requested to stay lying with eyes closed, without performing any task.

### Complex network metrics

For a detailed overview of complex network theory, we refer readers to refs 38, 41, 49. Each element of a network is known as a node (or vertex), and the relation between a pair of nodes is represented by a connecting link (or edge). Links can have weights associated with them and can be directed or undirected (or, equivalently bi-directional). Nodes connected by a single link are known as nearest neighbors^39^. Non-weighted undirected networks, i.e. those with symmetric and unweighted links are isomorphic to a binary symmetric matrix known as the adjacency matrix. When a pair of nodes *i* and *j* are neighbors, the adjacency matrix element is *a*_*i*,*j*_ = 1 and *a*_*i*,*j*_ = 0 otherwise^73, 74^. Standard quantities of interest that help to characterize the topology and complexity of networks^49, 53^ include node degree, geodesic distance, clustering coefficient, and local and global network efficiencies. We are interested here solely in non-weighted undirected networks.

Definitions:

(i) The degree *k*_*j*_ of a node *j* is the number of links that it has with other nodes. The degree distribution of a network is the normalized histogram of degrees over all nodes.
(ii) A geodesic path between two nodes is the shortest path from one to the other, assuming such a path exists. The geodesic distance *d*_*i*,*j*_ between nodes *i* and *j* is the number of links in the geodesic path. If there is no such path, the geodesic distance is defined as infinite. Given a network *G* with *N* nodes, the mean geodesic distance is given by
  1 $$D(G)=\frac{1}{N(N-\mathrm{1)}}\sum _{i\ne j}{d}_{i,j}.$$
(iii) The clustering coefficient quantifies the density of triads of linked nodes, e.g., the fraction of the neighbors of a node that are themselves neighbors. The clustering coefficient is defined by
  2 $$C(G)=\frac{1}{N}\sum _{i\ne j\ne h}\frac{2}{{k}_{i}({k}_{i}-\mathrm{1)}}{a}_{i,j}{a}_{j,h}{a}_{h,i},$$
  where *k*_*i*_ is the degree of node *i* and *a* is the adjacency matrix element.
(iv) The efficiency, typically defined as the reciprocal of the harmonic mean of geodesic distances, quantifies the influence of the topology on flux of information through the network. Efficiency can be global as well as local. We define global efficiency as

3 $${E}_{g}(G)=\frac{1}{N(N-\mathrm{1)}}\sum _{i\ne j\in G}\frac{1}{{d}_{i,j}},$$

and local efficiency as

4 $${E}_{l}(G)=\frac{1}{N}\sum _{i\in G}(\frac{1}{{n}_{i}({n}_{i}-\mathrm{1)}}\sum _{j\ne h\in {g}_{i}}\frac{1}{{d}_{h,j}}),$$

where *g*_*i*_ are the subnetworks formed by neighbors of node *i* and *n*_*i*_ is the number of nodes of this subnetwork^75^.

In addition to these standard network properties, we also use the Shannon entropy^76^ to quantify disorder or uncertainty. Specifically, we calculate the Shannon entropy functional of the distribution of node degrees. Let *P* be the normalized probability distribution for node degree *k*, i.e. ∑_*k*_ *P*(*k*) = 1. We define the Shannon entropy *S*[*P*] of the degree distribution *P*(*k*) for a network with *N* nodes by:

5 $$S[P]=-\sum _{k}P(k)\mathrm{log}\,P(k).$$

Often the logarithm of base 2 is used^77^ (e.g., in computer science), but we use the natural logarithm instead, so the entropy values shown are in natural information units rather than in bits.

### Maslov algorithm for generating randomized networks

Given *G*, one can select two non-overlapping pairs (*i*, *j*) and (*m*, *n*) of linked nodes, then unlink them, and cross-link the pairs according to (*i*, *m*) and (*j*, *n*). If this process is repeated many times, the links become randomized, but the degree of each node remains the same^56^. Hence the entropy of the degree distribution is also a conserved quantity.

### Calculation of correlation matrix for brain regions

We segmented the brain images into 110 brain regions according to the Harvard-Oxford cortical and subcortical structural atlas (threshold of >25%, using FMRIB Software Library, www.fmrib.ox.ac.uk/fsl). Six regions had to be excluded from further analysis, as they were not sampled for all subjects, due to technical limitations during image acquisition. For each of the 104 regions, an averaged fMRI time series was computed from all voxels (a voxel is a 3D image block, analogous to the 2D pixel). within that region using Marsbar (SPM toolbox). To reduce confounders, we applied a band-pass filter (≈0.03–0.07 Hz) using the maximum overlap wavelet transform (MODWT) with a Daubechies wavelet to divide the signal into 4 scales of different frequency bands. In keeping with the literature^4, 54^, that point that resting state typically leads to low frequency (≈0.01 to 0.1 Hz)^78^, we choose scale 3. We then calculated the Pearson correlation between these wavelet coefficients from all possible pairs, thus obtaining a 104 × 104 correlation matrix to represent each sample. We considered only correlation matrix elements that resulted in *p* < 0.05 in the hypothesis test. That is, we zero correlation matrix elements with non-reliable values (*p* ≥ 0.05).

### Construction of complex networks from fMRI images

A correlation matrix uniquely may define a weighted network. Nonetheless, we are interested in generating non-weighted networks. Hence, we need a function that maps correlation matrices to adjacency matrices. We use a thresholding function for this purpose. Given a correlation matrix, we obtain the adjacency matrix by applying a threshold to the absolute value of the elements of the correlation matrix. Specifically, if the absolute value of the correlation matrix element |*c*_*i*,*j*_| is larger than a defined threshold *η*, then a link is assumed and the adjacency matrix element is taken to be 1 (i.e., *a*_*i*,*j*_ = 1), while otherwise there is no link (*a*_*i*,*j*_ = 0). In order to obtain better statistics, we choose not a single value of *η* but a range of values instead. Then we analyze the behavior of the network properties over this range. Using this approach, we create a number of networks for each fMRI sample, all with the same number of nodes (104 nodes). For each of these networks, we choose *η* such that the density of links is the same before and after Ayahuasca intake.

We choose a range for the mean network degree to ensure the networks were fully connected but also relatively sparse. For this purpose, we adopt the following criteria: (i) the lowest correlation threshold (corresponding to maximum mean degree) must ensure that the networks have lower global efficiency and greater local efficiency than its randomized version. These criteria also ensure small-world behavior of the networks^79^ (according to the definition of Watts and Strogatz^59^). (ii) the upper correlation threshold (minimum mean degree) must ensure fully connected graphs and also guarantee that the networks obey the sparsity condition 〈*k*〉 > 2ln*N*, where *N* = 104 is the number of nodes. Since the quantity 2ln*N* never exceeds 10, our maximum threshold condition (minimum mean degree) was determined by the requirement for obtaining fully connected graphs. (A properly defined large *N* limit of this condition would be equivalent to the percolation threshold).

We choose common upper and lower thresholds for all correlation matrices (see Supplementary Fig. S3 for more details). These criteria are identical to those adopted by refs 4, 54. In order to obtain the same threshold range for all subjects, it was necessary to exclude two of them from the analysis, since there is no threshold range common between them and the other subjects. Data from a third subject was also excluded due to excessive head movement. Following the criteria described above, the threshold range is set to 0.25 ≤ *η* ≤ 0.43. We generate networks with mean degree in the range 24 ≤ 〈*k*〉 ≤ 39. We have shown in Figs 3, 5 and 6 network properties as a function of mean degree in increments of Δ〈*k*〉 = 1, for 16 different values of mean degree. We choose mean degree as the independent parameter instead of the correlation threshold because, unlike the latter, the mean degree is a network property. Despite the mean degree being a monotonic function of correlation threshold for all subjects, there is no *a priori* reason to expect universal behavior of network properties such as the entropy and geodesic distance (before vs. after Ayahuasca) as a function of the correlation threshold since the latter is not a network property (see Supplementary Fig. S3).

In summary, we have 7 human subjects suitable for both conditions (before and after ingestion). The resulting sets of networks allow 16 different comparisons (i.e. of differing mean degrees) for each subject before and after Ayahuasca ingestion. We calculate the topological measurements (using the Brain Connectivity Toolbox for Matlab^49^).

### Statistical testing

Comparisons between the two conditions (i.e., before and after Ayahuasca) are obtained from paired-sample Student’s *t*-tests. The *p*-values shown in Figs 3, 4, 5 and 6 are as follows: values *p* < 0.05 in bold and *p* < 0.005 indicated by asterisks (*). The implicitly assumed null hypothesis is that the difference of the paired values are normally distributed with zero mean.

## Electronic supplementary material

Supplementary discussion and figures
