# Supplementary material for: Shannon entropy of brain functional complex networks under the influence of the psychedelic Ayahuasca
Source: Sci Rep. 2017 Aug 7;7:7388. doi: 10.1038/s41598-017-06854-0 (PMC5547073; doi:10.1038/s41598-017-06854-0)
Supplement: Supplementary file 1 — Supplementary discussion and figures [file 41598_2017_6854_MOESM1_ESM.pdf]

Shannon entropy of brain functional complex networks under the influence of the psychedelic Ayahuasca

A. Viol[1,2,3,*], Fernanda Palhano-Fontes[4], Heloisa Onias[4], Draulio B. de Araujo[4], and G. M. Viswanathan[1,5]

[1] Department of Physics, Universidade Federal do Rio Grande do Norte, 59078-970 Natal–RN, Brazil
[2] Computational Biology Center, T. J. Watson Research Center, IBM, 10598 Yorktown Heights–NY, USA
[*] aline.viol@ufv.br
[4] Brain Institute, Universidade Federal do Rio Grande do Norte, 59078-970 Natal–RN, Brazil
[5] National Institute of Science and Technology of Complex Systems Universidade Federal do Rio Grande do Norte, 59078-970 Natal–RN, Brazil

Supplementary information

Supplementary discussion

Here we interpret our findings in the context of similarities between the use of Ayahuasca and mindfulness meditation and briefly discuss a plausible explanation that may underpin the possible therapeutic value of psychedelics. We also speculate about the meaning of our findings in relation to the distinction between primary vs. non-primary states of consciousness.

The idea that ordinary consciousness is not primary was previously put forth by Alan Watts[65], where he associated "primary consciousness" to what is nowadays termed mindfulness. When one is mindful, one pays attention to the experience of the present moment, letting go of ruminative thoughts and the tendency of wanting to predict the future based on memory of past events. It is not inconceivable that mindfuless may be related to the experience of "awakening" often described by users of psychedelics[19,20]. Watts described primary consciousness as follows[65]:

> The "primary consciousness," the basic mind which knows reality rather than ideas about it, does not know the future. It lives completely in the present, and perceives nothing more than what is at this moment. The ingenious brain, however, looks at that part of present experience called memory, and by studying it is able to make predictions. These predictions are, relatively, so accurate and reliable (e.g., "everyone will die") that the future assumes a high degree of reality — so high that the present loses its value.
>
> But the future is still not here, and cannot become a part of experienced reality until it is present. Since what we know of the future is made up of purely abstract and logical elements — inferences, guesses, deductions — it cannot be eaten, felt, smelled, seen, heard, or otherwise enjoyed. To pursue it is to pursue a constantly retreating phantom, and the faster you chase it, the faster it runs ahead. This is why all the affairs of civilization are rushed, why hardly anyone enjoys what he has, and is forever seeking more and more. Happiness, then, will consist, not of solid and substantial realities, but of such abstract and superficial things as promises, hopes, and assurances.

According to this view, ordinary consciousness of quotidian life is experienced by us not only directly in the present moment (i.e., by primary consciousness), but also through symbolic (hence, non-primary) mental representations that consist of "purely abstract and logical elements — inferences, guesses, deductions" or else of "abstract ... things as promises, hopes, and assurances." A key feature of this non-primary consciousness is that it is learned, e.g., one must first learn the abstract concept of "tomorrow" or "yesterday" in order to then think about them. These two perspectives of modes of consciousness compete with one another, in the sense that it is difficult to pay attention to what is happening now if you are actively thinking about what happened yesterday or what may happen tomorrow.

This view of consciousness leads to an interesting interpretation of our findings: the effects of Ayahuasca, and other psychedelics, may be due to the temporary removal of the some of the restrictions — hence learning — that are necessary for sustaining ordinary (adult trained) consciousness. Without these restrictions, the mind reverts to the more flexible state, in which self-referential narratives and thoughts about the past or the future are no longer experienced as identical to the reality that they are assumed to represent[65]. In the primary state, it is easy to directly perceive, through the immediate experience, that "the map is not the territory."

There is evidence of similar behaviors of brain functional networks when under the influence of Ayahuasca and during meditation[10,80]. It is not entirely surprising that psychedelics and meditative practices may have therapeutic potential in addiction and depression[26,81,82]. We speculate that in both cases, there is an increase in primary consciousness. It should be clear to the reader that mindfulness, which

is usually associated with meditative states[83], is closely related to the concept of primary consciousness. For example, mindfulness meditation could possibly be thought of as a method for the cultivation of the ability to more easily experience primary consciousness. Perhaps psychedelics exert their beneficial effects in a similar way.

Supplementary figures

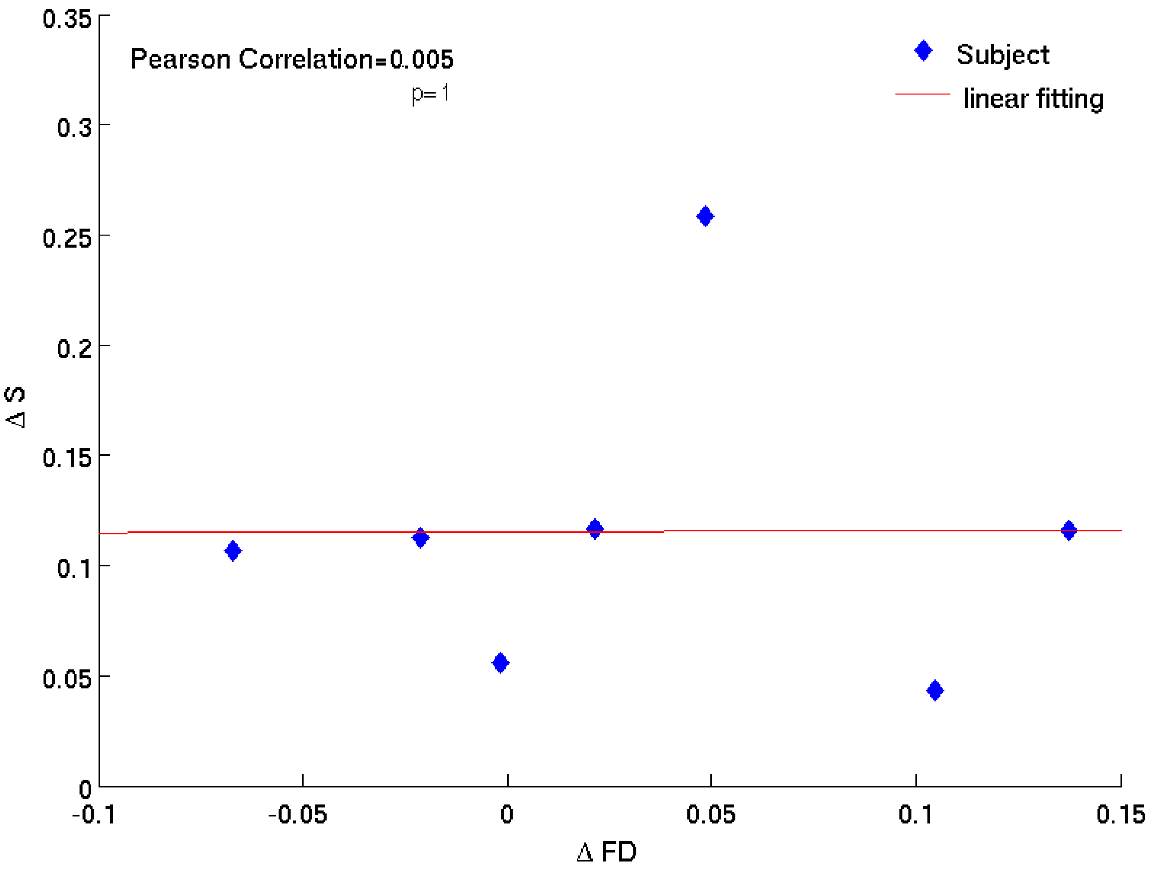

Supplementary Figure S1. Entropy increases not due to head motion. The plots show the variation of individual Frame Displacement (FD)[55] against individual variation in entropy for all subjects. We find no correlation between the Frame Displacement (FD) and increases in the Shannon entropy of the degree distributions before and after Ayauasca intake.

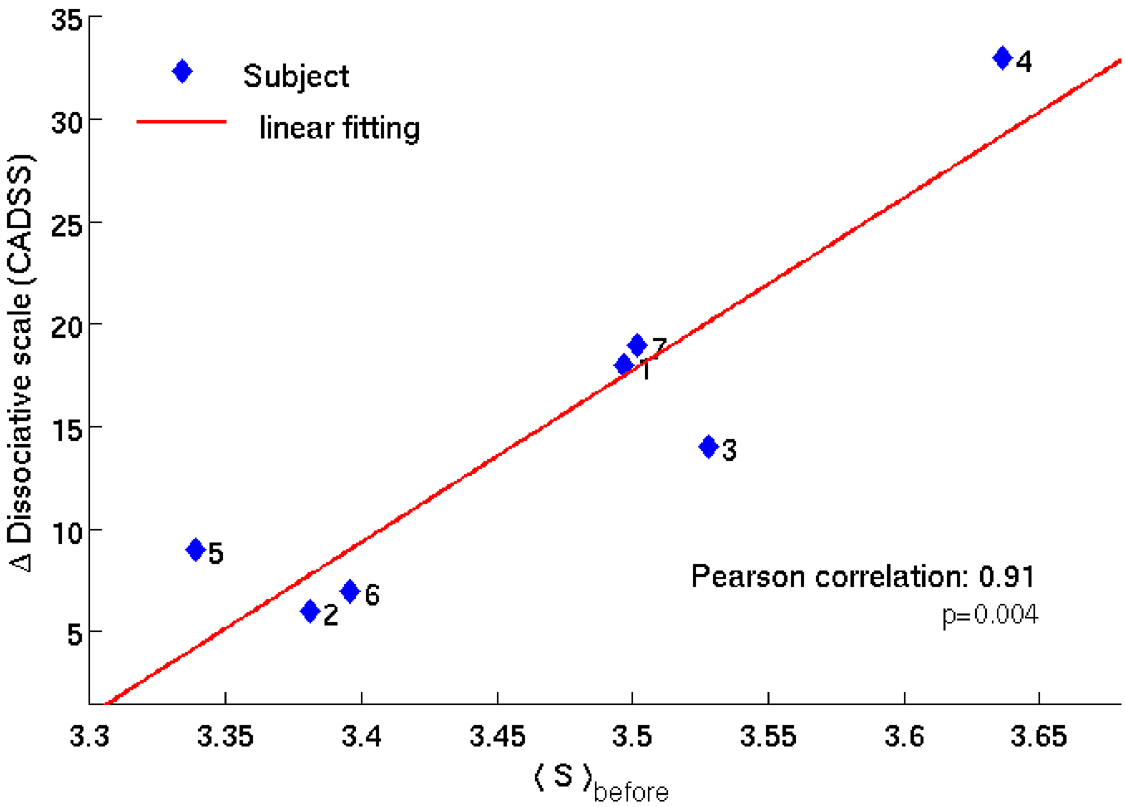

Supplementary Figure S2. Entropy before Ayahuasca ingestion is correlated with changes in dissociativity. The plot shows the entropy before Ayahuasca ingestion versus changes on the Clinician Administered Dissociative States Scale (CADSS)[57] score for all 7 subjects. We find a p-value of $p = 0.004$ for the remarkably large Pearson correlation $r = 0.91$. The limited number of subjects limits reliable conclusions. However, the unexpectedly strong correlation should encourage further investigations.

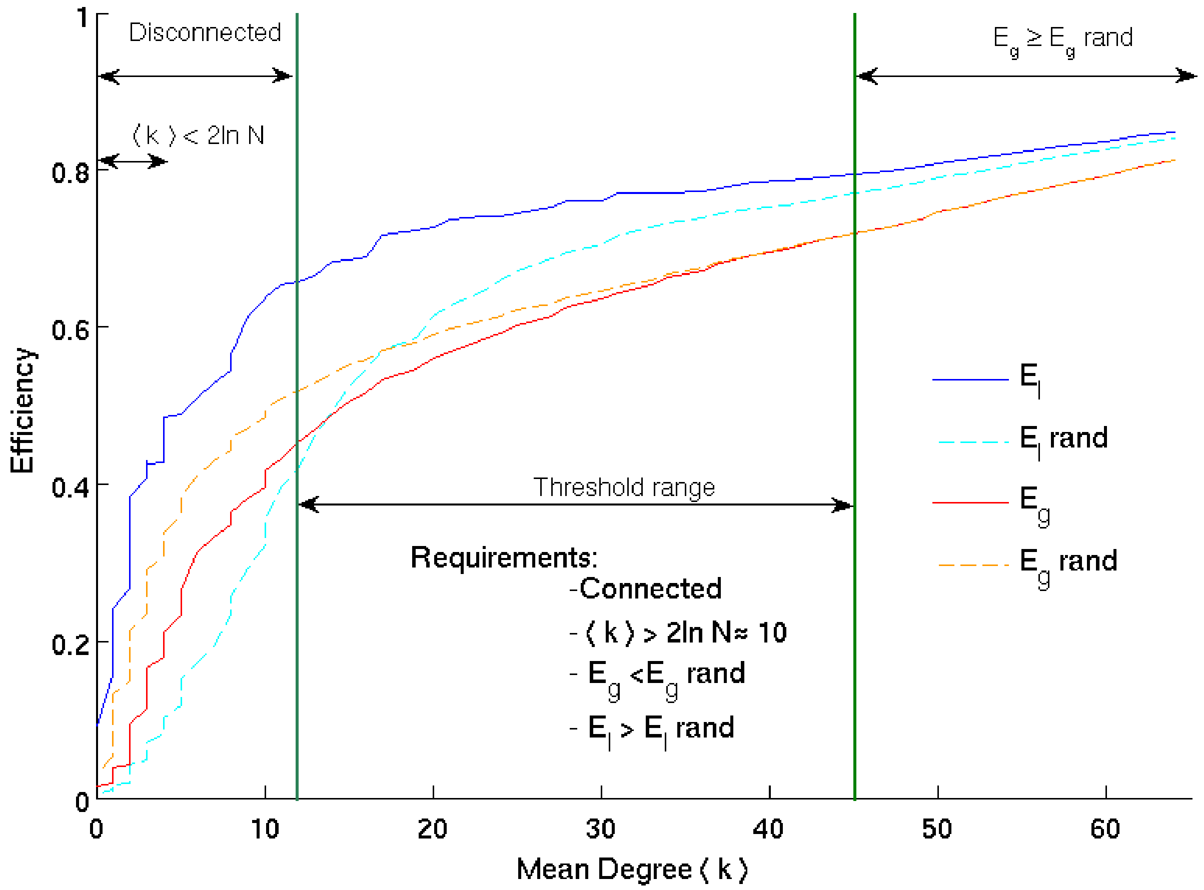

Supplementary Figure S3. Network efficiency and mean degree as a parametric function of correlation matrix thresholds for one of the samples. The range of thresholds that could be used for this particular sample is dilineated by the (green) vertical lines at $\langle k \rangle = 12$ and $\langle k \rangle = 45$. The upper threshold (for the lower mean degree) was set by two requirements: (i) the network must be sparse ($\langle k \rangle > 2\ln N$ and (ii) fully connected. The lower threshold (for upper mean degree) was set where both the global efficiency (E_g) is lower than the global efficiency ($E_g$rand) of the randomized version of the network and the local efficiency (E_l) is larger than the local efficiency (E_l) of the randomized version of the network. The final threshold range was defined considering a common range for all samples ($24 \leq k \leq 39$). We have followed the criteria used in ref. [54].

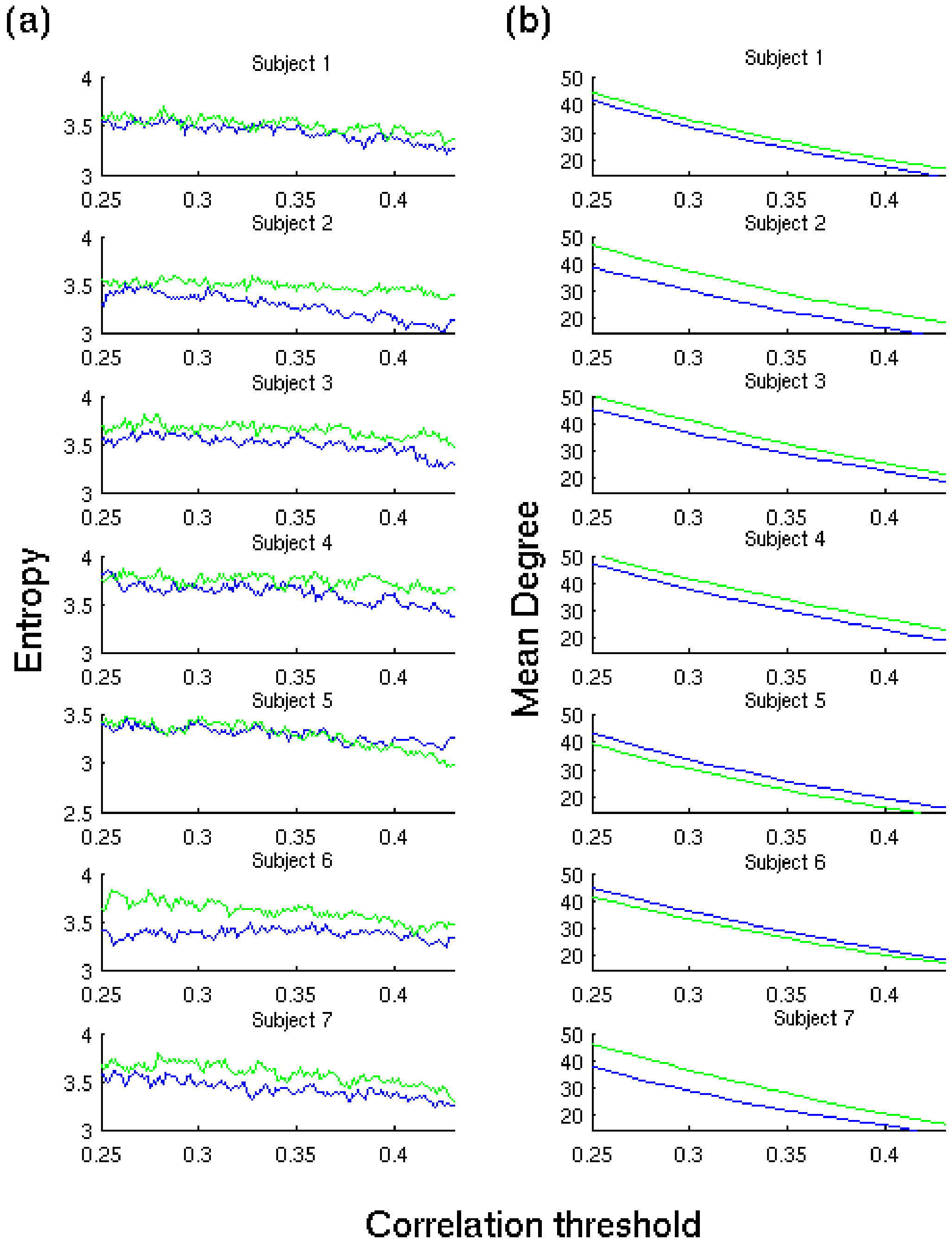

Supplementary Figure S4. Entropy and mean degree vs. correlation matrix thresholds. Column (a) shows the entropy and (b) the mean degree versus correlation thresholds for all 7 subjects. Although the mean degree is a smooth monotonic function of the threshold, the behavior before and after Ayahuasca intake is not identical for all 7 subjects (see subjects 5 and 6). Unlike the mean degree, the threshold is *not* a network property per se. The figure shows that the relative behavior before and after Ayahuasca of the mean degree and entropy as a function of the threshold varies among individuals, as expected. However, as shown in the main text, the entropy as a parametric function of the mean degree (parametrized by the threshold) shows nearly identical behavior for all subjects: the entropy increases after Ayahuasca ingestion.

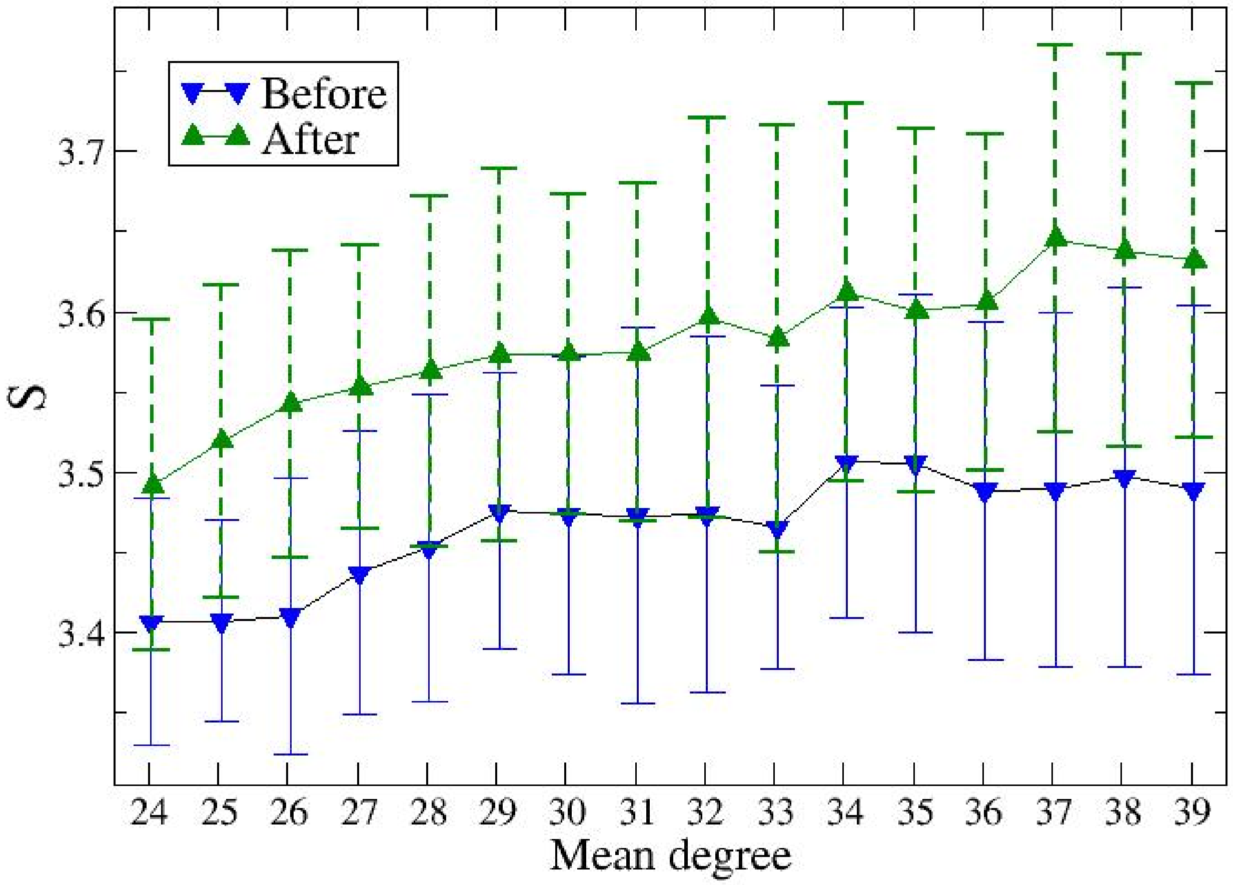

Supplementary Figure S5. Confidence intervals for entropy growth. Mean and 95% confidence intervals for the entropy of the distribution of node degrees calculated over all 7 subjects, as a function of mean degree, before (blue, solid interval bars) and after (green, dashed interval bars) Ayahuasca intake. Confidence intervals were estimated using the Matlab routines `paramci()` and `fitdist()`, assuming the normal distribution. Compare with Figure 3 of the main text.
